# Association Between *Mycobacterium tuberculosis* Sensitization and Insulin Resistance Among US Adults Screened for Type 2 Diabetes Mellitus

**DOI:** 10.1093/ofid/ofae568

**Published:** 2024-10-28

**Authors:** Itai M Magodoro, Aloice Aluoch, Brian Claggett, Moffat J Nyirenda, Mark J Siedner, Katalina A Wilkinson, Robert J Wilkinson, Ntobeko A B Ntusi

**Affiliations:** Department of Medicine, University of Cape Town, Observatory, Republic of South Africa; Piedmont Eastside Rheumatology, Snellville, Georgia, USA; Harvard Medical School, Boston, Massachusetts, USA; Department of Non-communicable Disease Epidemiology, London School of Hygiene & Tropical Medicine, London, UK; Harvard Medical School, Boston, Massachusetts, USA; Africa Health Research Institute, KwaZulu-Natal, South Africa; Centre for Infectious Diseases Research in Africa, Institute of Infectious Disease and Molecular Medicine, University of Cape Town, Observatory, Republic of South Africa; Francis Crick Institute, London, UK; Department of Medicine, University of Cape Town, Observatory, Republic of South Africa; Centre for Infectious Diseases Research in Africa, Institute of Infectious Disease and Molecular Medicine, University of Cape Town, Observatory, Republic of South Africa; Francis Crick Institute, London, UK; Department of Medicine, University of Cape Town, Observatory, Republic of South Africa; Centre for Infectious Diseases Research in Africa, Institute of Infectious Disease and Molecular Medicine, University of Cape Town, Observatory, Republic of South Africa; South African Medical Research Council, Tygerberg, Republic of South Africa; ARUA/Guild Cluster of Research Excellence on Noncommunicable Diseases and Associated Multimorbidity

**Keywords:** β-cell dysfunction, diabetes, insulin resistance, tuberculosis, *Mtb* sensitization

## Abstract

**Background:**

Type 2 diabetes mellitus (T2DM) may be a long-term sequela of infection with *Mycobacterium tuberculosis* (*Mtb*) by mechanisms that remain to be fully explained. We evaluated the association between *Mtb* sensitization and T2DM and, via mediation analysis, the extent to which it is mediated by insulin resistance and/or β-cell failure.

**Methods:**

Adults were assessed for T2DM by fasting plasma glucose, 2-hour oral glucose tolerance testing, and hemoglobin A_1c_; β-cell dysfunction and insulin resistance by homoeostasis model assessment 2; and *Mtb* sensitization by tuberculin skin testing. Associations between *Mtb* sensitization and T2DM were modeled with probit regression and decomposed into indirect effects of β-cell dysfunction and insulin resistance. Analyses were adjusted for sociodemographic, behavioral, and clinical characteristics.

**Results:**

We included 1843 adults. Individuals with *Mtb* sensitization were older than those without *Mtb* (median [IQR], 54 [39–64] vs 47 [33–62] years). As compared with being uninfected, *Mtb* sensitization was associated with T2DM (adjusted absolute risk difference, 9.34% [95% CI, 2.38%–15.0%]; *P* < .001) and increased insulin resistance (adjusted median difference, 0.16 [95% CI, .03–.29]; *P* = .014) but not β-cell dysfunction (adjusted median difference, −3.1 [95% CI, −10.4 to 4.3]; *P* = .42). In mediation analyses, insulin resistance mediated 18.3% (95% CI, 3.29%–36.0%; *P* = .020) of the total effect of the association between *Mtb* sensitization and T2DM.

**Conclusions:**

Definitive prospective studies examining incident T2DM following tuberculosis are warranted. Notwithstanding, our findings suggest that exposure to *Mtb* may be a novel risk factor for T2DM, likely driven by an increase in insulin resistance.

**Research in Context**

**What is already known about this subject?**

- Accumulating evidence suggests that prediabetes and new-onset type 2 diabetes mellitus (T2DM) may be long-term complications of exposure to *Mycobacterium tuberculosis* (*Mtb*) via mechanisms that remain to be unraveled.

**What is the key question?**

- To what extent do insulin resistance and β-cell failure mediate the association between *Mtb* sensitization and T2DM among US adults?

**What are the new findings?**

- *Mtb* sensitization is characterized by distinct glucose metabolic disturbances manifesting as increased risk of prevalent T2DM and isolated impaired fasting glucose.
- Insulin resistance, not β-cell impairment, likely mediates the observed diabetogenic effects of *Mtb* sensitization.

**How might this affect clinical and/or public health practice in the foreseeable future?**

- If corroborated by prospective studies, tuberculosis programs and individual clinical care must incorporate monitoring of serum glucose and long-term metabolic outcomes.
- This will be particularly urgent in sub-Saharan Africa and Southeast Asia, where scarce health resources coincide with overlapping endemic tuberculosis and epidemic T2DM.

Tuberculosis (TB) is a long-acknowledged complication of type 2 diabetes mellitus (T2DM) [1]. In recent times, however, T2DM has come to the fore as possibly a sequel of latent and active infection with *Mycobacterium tuberculosis* (*Mtb*) [2–4]. Heightened interest in the latter arises from the recognition that classical causes of T2DM, such as obesity, incompletely account for the disease's high incidence. This has prompted the search for novel diabetes risk factors, especially in low- and middle-income countries [5] and among indigenous and minority communities in high-income countries [6]. For example, diabetes in lean individuals (body mass index <25 kg/m^2^) accounts for 32% to 60% of cases of T2DM in sub-Saharan Africa and Southeast Asia [5, 7, 8]. This contrasts sharply with high-income countries, where at least 80% of patients with T2DM are overweight/obese (body mass index ≥25 kg/m^2^) [9]. With 120 million of the global 537 million prevalent T2DM cases (2021) [10], sub-Saharan Africa and Southeast Asia are coincidentally the seat of endemic TB [11]. Despite the significance of TB as a likely T2DM risk factor, the pathophysiologic mechanisms of this susceptibility remain unknown. These gaps imply that current clinical and public health strategies may be inadequate to prevent and control T2DM or mitigate the long-term consequences of TB.

The 2 final pathophysiologic pathways to T2DM development are islet β-cell failure and insulin resistance [9, 12]. Their causes are multiple and overlap. Among others, they include disordered inflammatory responses, lipid metabolism and gut microbiome for insulin resistance [9] and adverse early life exposures, oxidative stress, inflammatory cytokines, and amyloid deposition for β-cell failure [13]. Because T2DM is a heterogenous disease, the relative etiologic importance of β-cell failure and insulin resistance varies, as does their relative timing [12, 14, 15]. The contributory roles of these 2 key pathways to new-onset T2DM associated with mycobacterial infection remain to be elucidated. TB pancreatitis is uncommon while overt T2DM subsequent to it is very rare [16, 17]. However, *Mtb* infection likely has systemic-mediated diabetogenic effects [17]. For example, the proinflammatory cytokine cascade set off by *Mtb* antigens may drive insulin resistance in skeletal muscle, adipose tissue, and liver by adversely altering cellular and intracellular insulin signaling [17–19], while islet amyloid deposition associated with *Mtb* infection may precipitate β-cell failure [16, 18] through loss of β-cell mass and function [19]. Of note, amyloidosis in TB is documented in the United States [20], and TB is the commonest cause of secondary amyloidosis in low- and middle-income countries [21].

Therefore, detailed mechanistic studies of TB's diabetogenic potential remain an important and urgent priority. Here, we leveraged data from the 2011–2012 US National Health and Nutrition Examination Survey (NHANES) to address this challenge. Specifically, we assessed the association between *Mtb* sensitization and T2DM and evaluated the extent to which insulin resistance and β-cell failure are key mechanisms through which *Mtb* infection leads to higher T2DM risk.

## METHODS

We followed the STROBE guidelines (Strengthening the Reporting of Observational Studies in Epidemiology) in the conduct and reporting of our analyses [22].

### Study Design and Participants

Detailed descriptions of the NHANES are available elsewhere [23]. Briefly, the NHANES is a recurring series of biennial cross-sectional surveys of the noninstitutionalized US population with participants selected through multistage probability cluster sampling. Individual-level data on health status and its determinants are collected through questionnaires, physical examination, and laboratory testing. For the present study, we used data from the 2011–2012 NHANES cycle for the main analysis and the 1999–2000 NHANES cycle for the sensitivity analysis. All adults aged at least 20 years with complete data on fasting plasma glucose (FPG) and insulin, oral glucose tolerance testing, and tuberculin skin testing (TST) were eligible for inclusion in both analyses. Exclusion included any self-reported administration of insulin and/or oral hypoglycemic medications, previously diagnosed T2DM, and extreme values of fasting/prandial glucose (<3 or >25 mmol/L) or insulin (<20 or >300 pmol/L).

### Patient Consent Statement

Data collection and study procedures were approved by the Research Ethics Board of the US National Center for Health Statistics. This analysis of deidentified and publicly available data was deemed exempt from review by the University of Cape Town Human Research Ethics Committee.

### Study Covariates, Exposures, and Outcomes

#### Covariates

Data were extracted for participants' age, sex, race and ethnicity, socioeconomic status, alcohol use, blood pressure, waist circumference, serum cotinine, and past medical diagnoses including autoimmune conditions (asthma, psoriasis, celiac disease, arthritis, and thyroiditis). Race and ethnicity were self-reported as Hispanic, non-Hispanic White, non-Hispanic Black, and non-Hispanic Asian/other. The family poverty-to-income ratio (PIR) was used to assess socioeconomic status, with PIR ≤1.3 considered the poverty threshold [24]. We defined tobacco exposure as “none” if serum cotinine was <10 ng/mL, “passive exposure or light smoker” if ≥10 and <300 ng/mL, and “heavy smoker” if ≥300 ng/mL [25]. Participants who had no more than 12 alcohol-based drinks in the preceding year or ever and those who had at least 12 alcohol-based drinks in their lifetime but not in the past year were classified as “nondrinkers.” Those who had at least 12 drinks in the past year were defined as current drinkers and further classified as either “heavy current drinkers” if they reported ever having ≥4 drinks every day or “light/moderate current drinkers” if not [26]. Last, we extracted data on blood pressure and waist circumference.

#### *Mtb* Sensitization Status

*Mtb* sensitization was ascertained by TST with a tuberculin purified protein derivative product (Tubersol; Sanofi). Skin induration was measured 48 to 72 hours after intradermal placement of the purified protein derivative. Similar TST testing and quality control methods were followed in the 2 NHANES cycles [23]. Because neither chest radiographs nor TB symptoms screening was completed in the NHANES, skin induration ≥10 mm was considered indicative of *Mtb* sensitization [27]. Data on bacillus Calmette-Guérin (BCG) vaccination status were also not available.

#### Pancreatic Islet β-Cell Function and Insulin Resistance

Homoeostasis model assessment 2 (HOMA2) estimates of β-cell function (HOMA2-B) and insulin resistance (HOMA2-IR) were calculated by FPG and insulin with the HOMA calculator (University of Oxford) [28].

#### Diabetes Mellitus and Prediabetes States

The American Diabetes Association criteria (2023) were used to define glycemic status [29]. Diabetes mellitus was defined as any of the following: FPG ≥7.0 mmol/L, 2-hour oral glucose tolerance testing for plasma glucose (prandial plasma glucose [PPG]) ≥11.1 mmol/L, or hemoglobin A_1c_ (HbA_1c_) ≥6.5%. Data were not available to distinguish types 1 and 2 diabetes mellitus. Menke et al, using 1999–2000 NHANES data, estimated type 1 diabetes to be 4.8% of all diabetes in the United States [30]. Because we included participants aged ≥20 years and not currently using insulin, we therefore presumed all identified diabetes cases in our study to be T2DM. We tested the robustness of this assumption in sensitivity analyses that used a higher age cutoff (≥40 years) when the prevalence of undiagnosed type 1 diabetes is likely to be very low. For those without diabetes, we defined prediabetes as any of the following: HbA_1c_ ≥5.6% and <6.5%, FPG ≥5.6 and <7 mmol/L, or PPG ≥7.8 and <11.1 mmol/L. We similarly defined, among those without diabetes, isolated impaired fasting glucose (IFG) as FPG between 5.6 and 7.0 mmol/L and PPG <7.8 mmol/L and isolated impaired glucose tolerance as FPG <7.0 mmol/L and PPG between 7.8 and 11.1 mmol/L.

### Data Availability

The data that support the findings of this study are openly available in NHANES at https://www.cdc.gov/nchs/nhanes/index.htm.

### Data Analysis

Analyses were conducted with R version 3.6.3 (R Foundation for Statistical Computing) and Stata version 17.0 (StataCorp). All probability values were 2-sided, with *P* < .05 considered indicative of statistical significance. We did not apply sampling weights to our analyses. First, sociodemographic, behavioral, and clinical characteristics of the cohort were summarized by *Mtb* sensitization status. The distribution of participants' glucose metabolism indices were then plotted by *Mtb* sensitization status and their differences assessed by quantile regression models. The β coefficients (95% CI) from these models were reported as the difference in median values. Next, probit regression models with postestimation margins were applied to determine the association between *Mtb* sensitization and diabetes/prediabetes states, with results presented graphically. We additionally reported prevalence differences and prevalence ratios (PRs) of diabetes and prediabetes states by comparing those who were *Mtb* sensitized vs controls who were *Mtb* uninfected. These were determined from postestimation margins with linear combinations (for prevalence differences) or nonlinear combinations (for PRs) via the *mlincom* and *nlcom* commands of Stata version 18, respectively [31]. All regression models were adjusted for age, sex, race and ethnicity, family PIR, alcohol consumption, tobacco exposure, waist circumference, and self-reported autoimmunity.

The last step was mediation analysis via the counterfactual framework [32] to examine whether and how much insulin resistance or β-cell failure contributed to the association of *Mtb* sensitization with T2DM (Supplementary Figure 1). We used the *mediation* package in R [33], and analyses were performed by including 1 mediator at a time. Confounding factors for the mediation effect were the same as those included in the regression analyses. We modeled associations of *Mtb* sensitization based on the 50th percentile of insulin resistance or β-cell function using quantile regression (mediator model) and T2DM using probit regression (outcome model). Interactions were tested between mediators and exposure (*Mtb* sensitization–HOMA2-B and *Mtb* sensitization–HOMA2-IR) and, if statistically significant, were included in the mediation analyses.

### Sensitivity Analysis

The sensitivity analyses entailed 2 steps. First, we repeated the causal mediation analysis using the 2011–2012 NHANES cycle but including only participants aged ≥40 years. The second step, however, used the 1999–2000 NHANES cycle with similarly defined inclusion criteria (age ≥20 years old; not using insulin) and variables as the primary analysis.

## RESULTS

### Characteristics of Study Participants

**Figure 1.**
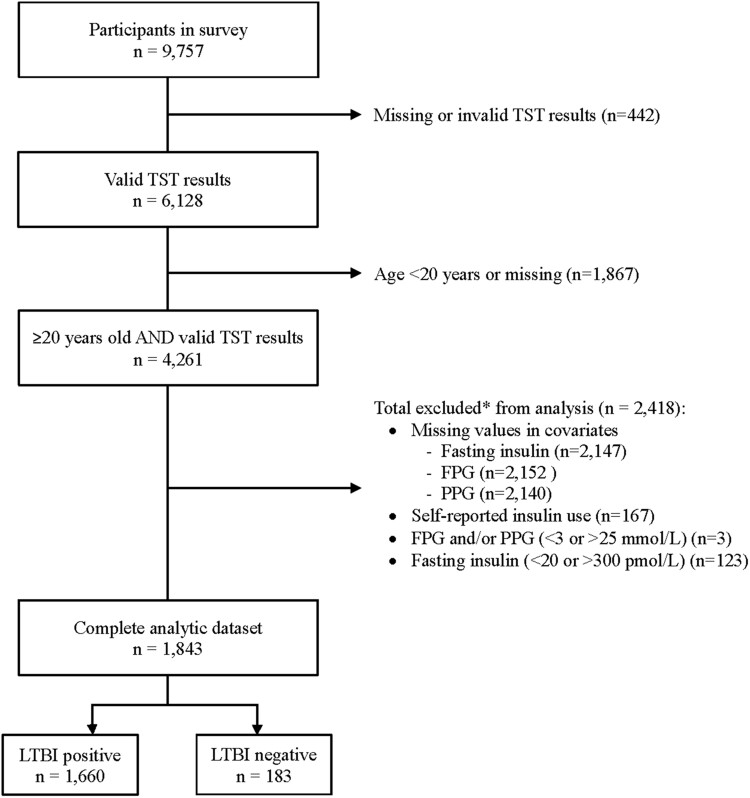
Participant inclusion in the final analytic sample based on the unweighted US NHANES 2011–2012 sample. FPG, fasting plasma glucose; LTBI, latent tuberculosis infection; NHANES, National Health and Nutrition Examination Survey; PPG, prandial plasma glucose; TST, tuberculin skin testing.

The analytic sample included 1843 adults (≥20 years old) with complete exposure (*Mtb* status) and outcomes data (T2DM, HOMA2 indices; Figure 1). Participants' baseline characteristics are summarized in Table 1. As compared with individuals who were uninfected, those with *Mtb* sensitization were older (median [IQR], 54 [39–64] vs 47 [33–62] years; *P* = .003) and more frequently of Hispanic race/ethnicity (42.6% vs 19.8%, *P* < .001) but less likely to use alcohol (55.7% vs 76.4%, *P* = .03). Rates of family poverty (PIR <1.3, 36.6% vs 34.7%; *P* = .064) and tobacco exposure (21.9% vs 22.5%, *P* = .98) were similar between the groups. There were also no notable differences in rates of self-reported prevalent comorbidities, although participants with *Mtb* sensitization had higher mean systolic blood pressure (125.1 vs 121.6 mm Hg, *P* = .010) than their counterparts without.

**Table 1.** Participants' Characteristics According to *Mtb* Sensitization Status, Unweighted US NHANES 2011–2012 Sample

	*Mtb* Sensitization Status		
Characteristic	Uninfected	Infected	*P* Value
Sample	1660 (90.1)	183 (9.9)	
Sociodemographic			
Age, y	47 (33–62)	54 (39–64)	.003
Male sex	817 (49.2)	100 (54.6)	.16
Race and ethnicity			<.001
Hispanic	328 (19.8)	78 (42.6)	
Non-Hispanic White	723 (43.6)	13 (7.1)	
Non-Hispanic Black	373 (22.5)	38 (20.8)	
Non-Hispanic Asian/other	236 (14.2)	54 (29.5)	
Family poverty-income ratio	2.0 (1.0–3.8)	1.8 (.9–3.5)	.064
Living below poverty threshold	576 (34.7)	67 (36.6)	.48
Behavioral			
Tobacco exposure			.98
None	1285 (77.5)	143 (78.1)	
Passive exposure or light smoker	282 (17.0)	30 (16.4)	
Heavy smoker	92 (5.5)	10 (5.5)	
Alcohol consumption			.030
Nondrinker	558 (33.6)	81 (44.3)	
Current, light/moderate drinker	872 (52.5)	83 (45.4)	
Current, heavy drinker	230 (13.9)	19 (10.4)	
Comorbidities			
Prevalent hypertension	666 (40.1)	81 (44.3)	.28
Systolic blood pressure, mm Hg^a^	121.6 (17.0)	125.1 (18.4)	.010
Diastolic blood pressure, mm Hg^a^	70.1 (12.4)	71.3 (13.0)	.21
Autoimmune disorders	272 (16.4)	22 (12.0)	.13
Prior major adverse cardiovascular events	157 (9.5)	21 (11.5)	.38
Chronic respiratory disorders	294 (17.7)	29 (15.8)	.53
Cancer/malignancy	136 (8.2)	8 (4.4)	.068

Values are presented as median (IQR) or No. (%). Percentages may not sum to 100% due to rounding.

Abbreviations: *Mtb*, *Mycobacterium tuberculosis*; NHANES, National Health and Nutrition Examination Survey.

^a^Mean (SD).

**Figure 2.**
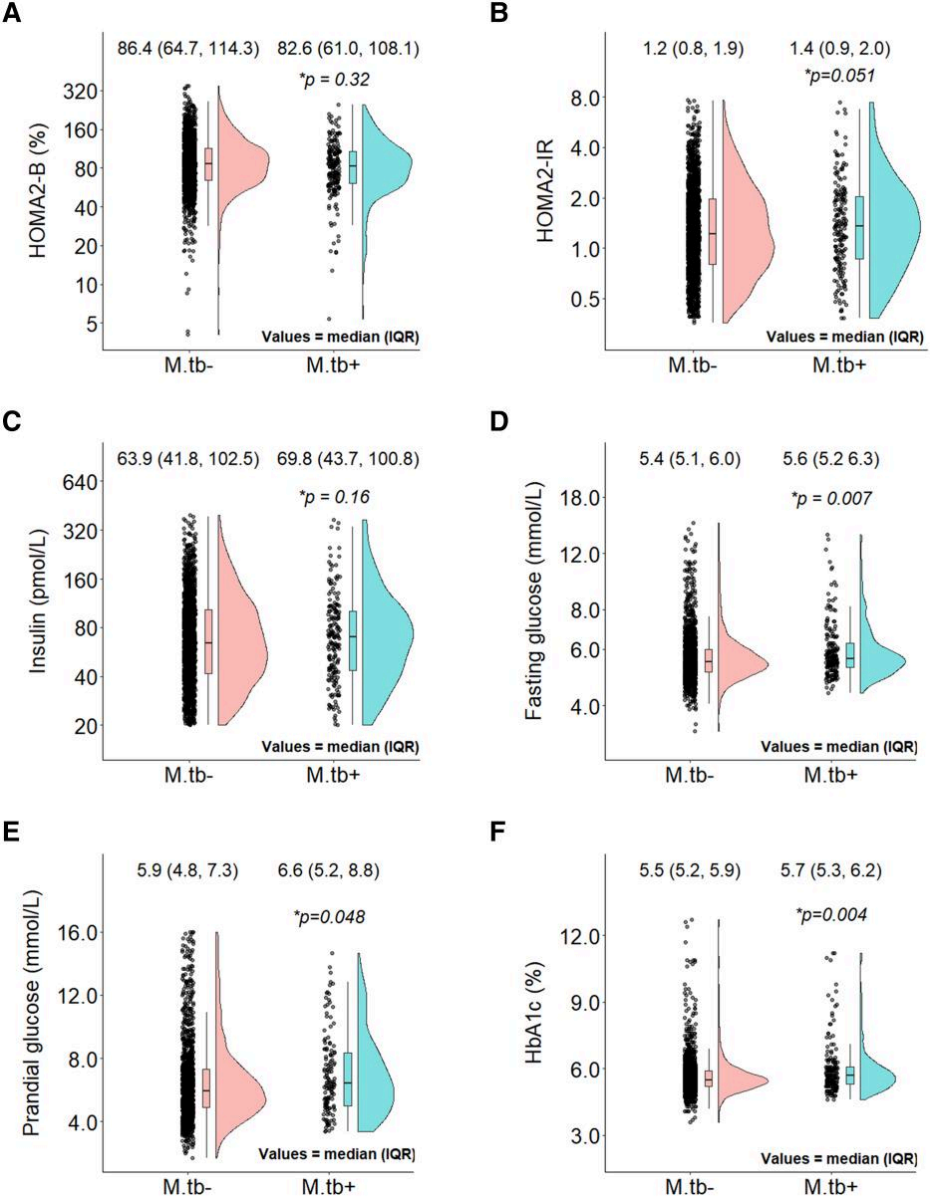
Participants' cardiometabolic characteristics according to *Mtb* sensitization status, per unweighted US NHANES 2011–2012 sample. *Mtb*+ and *Mtb*– represent *Mtb* sensitized and *Mtb* uninfected, respectively. Cardiometabolic characteristics: *A*, homoeostasis model assessment 2 of β-cell function (HOMA2-B); *B*, homoeostasis model assessment 2 of insulin resistance (HOMA2-IR); *C*, insulin; *D*, fasting glucose; *E*, prandial glucose; *F*, hemoglobin A_1c_ (HbA_1c_). *A–C*, The y-axes are scaled to log_10_. *P values derived from unadjusted quintile regression models of difference in median value (50th percentile). *Mtb*, *Mycobacterium tuberculosis*; NHANES, National Health and Nutrition Examination Survey.

Glucose metabolism profiles are summarized in Figure 2. *Mtb* sensitization was associated with higher FPG (median [IQR], 5.6 [5.2–6.3] vs 5.4 [5.1–6.0] mmol/L; *P* = .007; Figure 2*D*) and PPG (6.6 [5.2–8.8] vs 5.9 [4.8–7.3] mmol/L; *P* = .048; Figure 2*E*). Similarly, HbA_1c_ was higher with *Mtb sensitization* (5.7% [5.3%–6.2%]) than without (5.5% [5.2%–5.9%]; *P* = .004; Figure 2*F*).

### Distribution of Pancreatic Islet β-Cell Function, Fasting Plasma Insulin, and Insulin Resistance

The distribution of β-cell function, fasting plasma insulin, and insulin resistance is summarized in Figure 2 and Tables 2 and 3. The association between insulin resistance and *Mtb* sensitization was significant with and without confounder adjustment (Figure 2*B*). For example, the adjusted median difference (95% CI) in HOMA2-IR between the groups was +0.16 (+.03 to +.29, *P* = .014), being higher for those with *Mtb* sensitization. However, the were no significant *Mtb*-related differences in median β-cell function (adjusted median difference, −3.1 [95% CI, −10.5 to +4.3]; *P* = .42). In addition to *Mtb* sensitization, the other significant determinants of increased insulin resistance were female (vs male) sex, older age, greater central adiposity, and alcohol abstention. In contrast, male (vs female) sex, older age, and living in poverty were significantly correlated with impaired β-cell impairment.

**Table 2.** Associations Between *Mtb* Sensitization and Insulin Resistance, Unweighted US NHANES 2011–2012 Sample

	Difference in Median HOMA2-IR^a^			
Characteristic	Unadjusted	*P* Value	Adjusted^b^	*P* Value
*Mtb* sensitization status				
Uninfected	Reference		Reference	
Sensitized	+0.14 (−.01, +.22)	.051	+0.16 (+.03, +.29)	.014
Sex				
Female	Reference		Reference	
Male	+0.02 (−.08, +.12)	.41	−0.16 (−.24, −.08)	<.001
5-y age increase	+0.02 (+.01, +.03)	.001	+0.03 (+.01, +.04)	<.001
5-cm waist circumference increase	+0.15 (+.14, +.17)	<.001	+0.17 (+.16, +.17)	<.001
Race and ethnicity				
Hispanic	Reference		Reference	
Non-Hispanic White	−0.20 (−.35, −.05)	.011	−0.12 (−.24, −.02)	.016
Non-Hispanic Black	−0.01 (−.19, +.17)	.92	+0.05 (−.06, +.16)	.87
Non-Hispanic Asian/other	−0.33 (−.49, −.19)	<.001	+0.09 (−.05, +.22)	.20
Family poverty-income ratio				
Nonpoor	Reference		Reference	
Living below poverty threshold	+0.15 (+.02, +.28)	.019	+0.04 (−.05, +.13)	.89
Tobacco exposure				
None	Reference		Reference	
Passive exposure or light smoker	+0.01 (−.13, +.13)	.91	+0.03 (−.08, +.14)	.58
Heavy smoker	−0.20 (−.41, +.01)	.057	−0.01 (−.18, +.17)	.96
Alcohol consumption				
Nondrinker	Reference		Reference	
Current light/moderate drinker	−0.23 (−.32, −.14)	<.001	−0.14 (−.23, −.06)	.001
Current heavy drinker	−0.09 (−.22, +.04)	.19	−0.17 (−.31, −.03)	.007
Autoimmune disorders				
None	Reference		Reference	
Self-reported disease	+0.14 (+.05, +.23)	.004	−0.01 (−.16, +.18)	.98

Data are presented as mean (95% CI).

Abbreviations: HOMA2-IR, homeostatic model assessment 2 of insulin resistance; *Mtb*, *Mycobacterium tuberculosis*; NHANES, National Health and Nutrition Examination Survey.

^a^β Coefficients (95% CI) from quantile regression models reported as difference in median value.

^b^Model adjusted for sex, age, race and ethnicity, family poverty-income ratio, alcohol consumption, tobacco exposure, waist circumference, and self-reported autoimmunity.

**Table 3.** Associations Between *Mtb* Sensitization and Pancreatic β-Cell Function, Unweighted US NHANES 2011–2012 Sample

	Difference in Median HOMA2-B^a^			
Characteristic	Unadjusted	*P* Value	Adjusted^b^	*P* Value
*Mtb* sensitization status				
Uninfected	Reference		Reference	
Sensitized	−3.8 (−9.6, +2.0)	.20	−3.1 (−10.5, +4.3)	.42
Sex				
Female	Reference		Reference	
Male	−9.9 (−14.3, −5.5)	<.001	−11.3 (−14.5, −8.0)	<.001
5-y age increase	−2.4 (−3.2, −1.7)	<.001	−3.2 (−3.0, −2.7)	<.001
5-cm waist circumference increase	+4.5 (+3.9, +5.2)	<.001	+5.4 (+4.9, +5.9)	<.001
Race and ethnicity				
Hispanic	Reference		Reference	
Non-Hispanic White	−2.3 (−7.5, +2.9)	.38	+.7 (−3.3, +4.7)	.76
Non-Hispanic Black	+3.3 (−2.9, +9.5)	.32	+4.6 (−1.4, +10.5)	.13
Non-Hispanic Asian/other	−8.7 (−14.1, −3.2)	.002	+1.3 (−3.2, +5.8)	.57
Family poverty-income ratio				
Nonpoor	Reference		Reference	
Living below poverty threshold	+2.1 (−2.7, +6.9)	.38	−3.7 (−6.9, −.5)	.024
Tobacco exposure				
None	Reference		Reference	
Passive exposure or light smoker	−1.2 (−7.3, +4.9)	.69	−1.7 (−6.9, +3.3)	.51
Heavy smoker	−7.8 (−18.6, +2.9)	.64	−5.5 (−12.0, +1.1)	.11
Alcohol consumption				
Nondrinker	Reference		Reference	
Current light/moderate drinker	−3.7 (−8.6, +1.2)	.15	−3.6 (−8.1, +.8)	.39
Current heavy drinker	−4.4 (−12.6, +3.8)	.29	−3.2 (−10.5, +4.1)	.39
Autoimmune disorders				
None	Reference		Reference	
Self-reported disease	+1.7 (−4.3, +7.7)	.58	+.4 (−4.4, +5.1)	.51

Data are presented as mean (95% CI).

Abbreviations: HOMA2-B, homeostatic model assessment 2 of β-cell function; *Mtb*, *Mycobacterium tuberculosis*; NHANES, National Health and Nutrition Examination Survey.

^a^β Coefficients (95% CI) from quintile regression models reported as difference in median value.

^b^Model adjusted for sex, age, race and ethnicity, family poverty-income ratio, alcohol consumption, tobacco exposure, waist circumference, and self-reported autoimmunity.

### Prevalence of Diabetes and Prediabetes States

**Figure 3.**
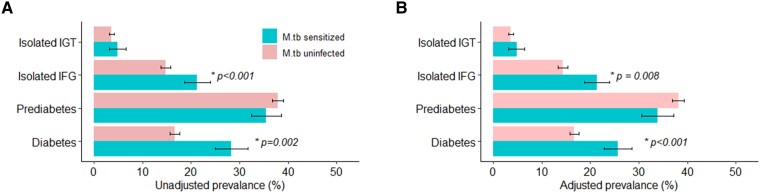
Diabetes mellitus and prediabetic states according to *Mtb* sensitization status, per unweighted US NHANES 2011–2012 sample: *A*, unadjusted prevalence; *B*, adjusted prevalence. Error bars indicate 95% CIs. *P values are for *Mtb-*sensitized vs *Mtb-*uninfected comparisons and are shown only if statistically significant (*P* < .05). Prevalence adjusted for sex, age, race and ethnicity, family poverty-income ratio, alcohol consumption, tobacco exposure, waist circumference, and self-reported autoimmunity. Prediabetes defined among those without diabetes as any one of the following: hemoglobin A_1c_ ≥5.6% and <6.5%, fasting plasma glucose ≥5.6 and <7 mmol/L, or prandial plasma glucose ≥7.8 and <11.1 mmol/L. IFG, isolated impaired fasting glucose; IGT, impaired glucose tolerance; *Mtb*, *Mycobacterium tuberculosis*; NHANES, National Health and Nutrition Examination Survey.

T2DM was significantly more prevalent with *Mtb* sensitization as compared with being *Mtb* uninfected in unadjusted comparisons (28.4% vs 16.6%, *P* = .002; Figure 3*A* and Supplementary Table 1) and adjusted (25.7% vs 16.7%, *P* < .001; Figure 3*B*). This corresponded to a 1.5-fold higher T2DM prevalence with *Mtb* sensitization vs being uninfected (unadjusted PR, 1.71 [95% CI, 1.27–2.15]; *P* < .001; adjusted PR, 1.54 [95% CI, 1.15–1.92]; *P* < .001). Among the prediabetic states, only isolated IFG differed significantly by *Mtb* status. It was more prevalent among the *Mtb* sensitized (adjusted prevalence, 21.4% [95% CI, 19.4%–23.6%]) than uninfected (14.3% [9.2%–19.4%], *P* = .008]. Participants with *Mtb* sensitization were as likely to have either prediabetes (adjusted PR, 0.89 [95% CI, .71–1.07]; *P* = .246) or isolated impaired glucose tolerance (1.34 [.42–2.26], *P* = .455) as those who were *Mtb* uninfected.

### Mediated Effects of Islet β-Cell Function and Insulin Resistance

Exposure-mediator interactions (*Mtb* sensitization–HOMA2-B, *P* = .43; *Mtb* sensitization–HOMA2-IR, *P* = .97) were not statistically significant and thus not included in the mediation models. As compared with being uninfected, *Mtb* sensitization was associated with a greater risk of prevalent T2DM (adjusted absolute risk difference [ARD], 9.34% [95% CI, 2.38%–15.0%]; *P* < .001; Table 4). Of this total effect, 18.3% (95% CI, 3.29%–36.0%)—corresponding to an adjusted ARD of 1.65% (.31%–3.0%)—was due to mediation by insulin resistance. In contrast, the pathway via β-cell function was not significant (adjusted ARD, 0.57% [95% CI, −.87% to 2.0%]; *P* = .48); thus, β-cell function did not meaningfully contribute to an association between *Mtb* sensitization and T2DM (proportion mediated, 6.33% [95% CI, −10.8% to 21.0%]; *P* = .50). The magnitude and direction of these findings were replicated when the mediation analysis was repeated with either the 1999–2000 NHANES cycle or the 2011–2012 NHANES cycle but with higher inclusion age (≥40 vs ≥20 years).

**Table 4.** Mediated Effects of Insulin Resistance and β-Cell Function in the Association Between *Mtb* Sensitization and Diabetes Mellitus, Unweighted US NHANES 2011–2012 Sample

	Adjusted Absolute Risk Difference, % (95% CI)^a^						Proportion Mediated	
Mediator	Total Effect	*P* Value	Direct Effect	*P* Value	Indirect Effect	*P* Value	% (95% CI)	*P* Value
HOMA2-IR								
Primary analysis								
NHANES 2011–2012; age ≥20 y	9.34 (2.38, 15.0)	<.001	7.68 (1.43, 12.0)	<.001	1.65 (.31, 3.00)	.020	18.3 (3.29, 36.0)	.020
Sensitivity analysis								
NHANES 2011–2012; age ≥ 40 y	10.8 (7.42, 22.0)	<.001	9.31 (4.86, 19.0)	<.001	1.62 (1.45, 3.00)	<.001	13.5 (11.4, 38.0)	<.001
NHANES 1999–2000	10.7 (3.5, 18.6)		8.9 (2.0, 16.1)		1.8 (.4, 3.4)		16.7 (9.4, 48.7)	
HOMA2-B								
Primary analysis								
NHANES 2011–2012; age ≥20 y	9.04 (4.60, 16.0)	<.001	8.47 (4.58, 14.0)	<.001	.57 (−.87, 2.00)	.481	6.33 (−10.8, 21.0)	.501
Sensitivity analysis								
NHANES 2011–2012; age ≥ 40 y	11.6 (4.67, 19.0)	<.001	11.5 (4.30, 19.0)	<.001	.0 (−1.4, 3.00)	.562	.89 (−.96, 22.0)	.560
NHANES 1999–2000	12.5 (4.6, 20.6)		11.9 (4.9, 19.4)	…	.5 (−2.1, 3.3)	…	4.5 (2.8, 12.3)	…

Primary analysis based on 2011–2012 NHANES cycle with inclusion age ≥20 years. Sensitivity analyses based on 2011–2012 NHANES cycle with inclusion age ≥40 years and 1999–2000 NHANES cycle with inclusion age ≥20 years.

Abbreviations: HOMA2-B, homeostatic model assessment 2 of β-cell function; HOMA2-IR, homeostatic model assessment 2 of insulin resistance; *Mtb*, *Mycobacterium tuberculosis*; NHANES, National Health and Nutrition Examination Survey.

^a^Adjusted for sex, age, race and ethnicity, family poverty-income ratio, alcohol consumption, tobacco exposure, waist circumference, and self-reported autoimmunity. Absolute risk difference between *Mtb* sensitized and *Mtb* uninfected as reference group.

## DISCUSSION

We found that *Mtb* sensitization in US adults is characterized by distinct glucose metabolic disturbances (T2DM and isolated IFG) independent of age, sex, and other potential determinants. In tandem, we also observed fasting and prandial hyperglycemia as well as elevated HbA_1c_ with *Mtb* sensitization. Insulin resistance, not β-cell impairment, mediated the observed diabetogenic effects of *Mtb* sensitization. Because our study was cross-sectional, we make these inferences with caution. Definitive prospective mechanistic studies of incident T2DM following *Mtb* exposure are required. Notwithstanding, our findings do suggest that sharpened focus on long-term metabolic outcomes of TB is warranted. This is particularly urgent in sub-Saharan Africa and Southeast Asia, where scarce health resources must meet increasingly overlapping endemic TB and epidemic T2DM.

Exposure to *Mtb* represents an immunopathologic spectrum [34]. At one end is asymptomatic immune sensitization to mycobacterial antigens, as evidenced by reactive TST and/or interferon γ release assays in apparently healthy persons. Clinically overt and oftentimes fatal TB disease lies at the opposite pole. How T2DM risk varies along this spectrum is unknown. Whereas we investigated impaired glucose regulation in *Mtb* sensitization, most studies to date have focused on active TB and report glucose intolerance in up to half of the cases (16.5%–49%) [35]. The features of impaired glucose regulation associated with *Mtb* sensitization in our study were fasting and marginally prandial hyperglycemia and, in turn, isolated IFG. According to the literature, the majority of patients with dysglycemia during active TB regress to normoglycemia after anti-TB treatment [17]. This transient hyperglycemia is seen with other severe systemic infections, such as community-acquired pneumonia, supporting the possibility of a nonspecific stress response to infection [36]. Unfortunately, our study lacked follow-up data on the trajectories of *Mtb*-associated impaired glucose regulation. It is important to note, however, that these preclinical metabolic states independently predict future new-onset T2DM in the general population [9].

A drawback of most available studies is the fact that their cross-sectional design is compatible with undetected T2DM being present prior to the onset of TB. Recognizing this bidirectionality of the TB-T2DM relationship [2], we nevertheless proceeded from the hypothesis that *Mtb* sensitization was diabetogenic. Our mediation analyses followed the counterfactual framework that provides valid causal estimates, subject to necessary assumptions of positivity, consistency, and no unmeasured confounding for the exposure-outcome, mediator-outcome, and exposure-mediator relationships [32]. The total effect of exposure on outcome is decomposed into direct and indirect effects through a mediator. The direct effect captures the effect of exposure on outcome if the path via mediators is prevented or removed hypothetically. Our finding that *Mtb* sensitization was a risk factor for T2DM is thus consistent with the available, albeit limited, prospective studies of new-onset T2DM following *Mtb* exposure. Pearson et al [4] reported a 5-fold higher T2DM incidence among individuals with a history of active TB using UK-wide primary care data, while Magee et al [37] found that US veterans with reactive TST and/or interferon γ release assay had up to 1.3 times higher risk of new-onset T2DM than their peers who were nonreactive. Yet, Young et al found no evidence of increased post-TB T2DM risk in a prospective cohort in Oxford, England [38].

From a mechanistic standpoint, studies in humans and animals provide evidence for the diabetogenicity of *Mtb* [17]. For example, in the murine model of TB, *Mtb* has been shown to cause insulin resistance via dysregulation of lipid metabolism with ectopic deposition of fat in the liver and skeletal muscles [39]. Insulin resistance has also been attributed to impaired liver function due to the toxic effect of anti-TB drugs [17]. Inflammation from *Mtb* infection results in an environment of sustained proinflammatory cytokine production, often leading to metabolic dysregulation and eventually insulin resistance [17]. Philips et al in South Africa found insulin resistance in 25% of patients with newly diagnosed TB [40]. Similarly, we found *Mtb* sensitization to be associated with greater insulin resistance, which in turn was on the mechanistic pathway to T2DM. In contrast, we did not find evidence for a role for β-cell impairment. The putative link between exposure to *Mtb* and β-cell impairment will be pancreatic amyloid deposition leading to loss of islet mass and function [16, 18]. In fact, participants who were *Mtb* sensitized in our study had lower HOMA2-B than their counterparts who were uninfected, although the differences did not reach statistical significance. This could be attributed, at least in part, to our exclusion of participants taking insulin, some of whom could have significant insulin deficiency.

### Strengths and Limitations

Our study is among the first to explore the mechanistic roles of islet β-cell failure and insulin resistance in the diabetogenicity of *Mtb* and thus sheds novel insights into a challenge of growing clinical and public health concern. The NHANES samples are drawn to reflect the diversity of the US population. As compared with currently available studies that are mostly health facility based, our results have greater generalizability. Data on TB-related symptoms, chest radiographs, and sputum examinations in conjunction with TST would have enabled better stratification of *Mtb* sensitization into those who have eliminated, controlled, and subclinical TB infection [34]. Our use of TST alone means likely false-positive results due to sensitization by environmental mycobacteria and/or BCG. Unfortunately, we did not have BCG vaccination data or the ability to robustly distinguish type 1 diabetes from T2DM.

The cross-sectional design of our study remains a concerning limitation. Whereas we hypothesized that *Mtb* sensitization led to diabetes, the bidirectionality of the TB-T2DM relationship means that the cross-sectional association that we observed could be contributed by both directions. However, some considerations do mitigate against this. First, our assumption of the “unidirectional” association is not biologically implausible, as it is supported by a growing body of evidence, some of it reviewed here. Indeed, our results, resting as they do on this “unidirectional assumption,” are consistent with this broader evidence. Next, our exclusion of participants with self-reported T2DM or use of insulin or oral hypoglycemic medications likely curtailed the inclusion of long-standing T2DM. Last, the consistency of our primary analysis (NHANES 2011–2012) and sensitivity analysis (NHANES 1999–2000) does give some reassurance.

## CONCLUSION

Definitive prospective studies examining incident T2DM following *Mtb* exposure are urgently required, especially in sub-Saharan Africa and Southeast Asia. Future studies should aim for a more granular definition of *Mtb* sensitization by using imaging, laboratory testing, and clinical examination, as well as more accurate definitions of T2DM. Notwithstanding, our findings suggest that exposure to *Mtb* may be a novel risk factor for T2DM, likely driven by an increase in insulin resistance.

## Supplementary Material

ofae568_Supplementary_Data

## Supplementary Data

Supplementary materials are available at *Open Forum Infectious Diseases* online. Consisting of data provided by the authors to benefit the reader, the posted materials are not copyedited and are the sole responsibility of the authors, so questions or comments should be addressed to the corresponding author.

## Notes

***Acknowledgments.*** For the purposes of open access, the authors have applied a CC-BY public copyright to any author-accepted manuscript arising from this submission.

***Author contributions*.** I. M. M.: data curation, project administration and validation, visualization, writing–original draft. I. M. M. and B. C.: formal analysis. I. M. M., A. A., R. J. W., and N. A. B. N.: conceptualization. I. M. M., A. A., B. C., M. J. N., M. J. S., K. A. W., R. J. W., and N. A. B. N.: investigation, methodology, and writing–review and editing.

***Financial support*.** There was no funding source for this study. R. J. W. and K. A. W. are funded by the Francis Crick Institute, which is supported by Cancer Research UK (FC2112), Medical Research Council (UKRI) (FC2112), and Wellcome Trust (203135) (FC2112). M. J. S. receives support from the US National Institutes of Health (K24 HL166024). N. A. B. N. gratefully acknowledges support from the South African Medical Research Council, National Research Foundation, the US National Institutes of Health, Medical Research Council (UKRI), and the Lily and Ernst Hausmann Trust.
