# Supplementary material for: Association Between Mycobacterium tuberculosis Sensitization and Insulin Resistance Among US Adults Screened for Type 2 Diabetes Mellitus
Source: Open Forum Infect Dis. 2024 Oct 28;11(10):ofae568. doi: 10.1093/ofid/ofae568 (PMC11518572; doi:10.1093/ofid/ofae568)
Supplement: ofae568_Supplementary_Data [file ofae568_supplementary_data.zip › Supplementary Material.docx]

**Supplementary Material**

**Supplementary Figure 1. Directed acyclic graph (DAG) of the association to *M.tb* sensitization and diabetes mellitus**.

* covariates = sex, age, race/ethnicity, family poverty-ratio ratio (PIR), alcohol consumption, tobacco exposure, waist circumference, and self-reported auto-immunity.

**Supplementary Table 1. Unadjusted and adjusted prevalence, prevalence difference and prevalence ratio of diabetes mellitus and prediabetic states according to M.tb sensitization status, unweighted US NHANES 2011-2012 sample.**

| **Glucose status** | **Prevalence^α^** | | **Prevalence**  **difference^α,^** ^β^ | **P value** | **Prevalence**  **ratio^α,^** ^β^ | **P value** |
| --- | --- | --- | --- | --- | --- | --- |
|  | **M.tb uninfected** | **M.tb sensitized** |  |  |  |  |
| Diabetes |  | | | | | |
| *Unadjusted* | 16.6 (14.8, 18.3) | 28.4 (21.7, 35.1) | +11.8 (+4.9, +18.7) | <0.001 | 1.71 (1.27, 2.15) | <0.001 |
| *Adjusted* | 16.7 (14.9, 18.6) | 25.7 (20.1, 31.3) | +9.0 (+3.0, +15.1) | 0.003 | 1.54 (1.15, 1.92) | <0.001 |
| Prediabetes |  | | | | | |
| *Unadjusted* | 38.0 (35.7, 40.2) | 35.5 (29.4, 41.6) | -2.4 (-8.7, +3.9) | 0.445 | 0.94 (0.77, 1.10) | 0.445 |
| *Adjusted* | 38.1 (35.8, 40.3) | 34.0 (27.4, 40.5) | -4.1 (-11.4, +2.8) | 0.246 | 0.89 (0.71, 1.07) | 0.246 |
| Isolated IFG |  | | | | | |
| *Unadjusted* | 14.8 (9.4, 20.1) | 21.3 (19.4, 23.3) | +6.6 (+1.0, +12.3) | 0.025 | 1.45 (1.01, 1.98) | 0.025 |
| *Adjusted* | 14.4 (9.4, 19.4) | 21.4 (19.4, 23.4) | +7.0 (+1.5, +12.6) | 0.013 | 1.48 (1.04, 2.03) | 0.013 |
| Isolated IGT |  | | | | | |
| *Unadjusted* | 3.6 (2.7, 4.5) | 4.9 (1.7, 8.1) | +1.3 (-1.9, +4.5) | 0.429 | 1.36 (0.45, 2.27) | 0.429 |
| *Adjusted* | 3.6 (2.7, 4.4) | 4.8 (1.6, 8.0) | +1.2 (-2.0, +4.4) | 0.455 | 1.34 (0.42, 2.26) | 0.455 |

^α^ Where adjusted, prevalence, prevalence difference and prevalence ratio adjusted for sex, age, race/ethnicity, family poverty-income ratio, alcohol consumption, tobacco exposure, waist circumference, and self-reported auto-immunity.

^β^ M.tb uninfected is the reference group.

Prediabetes = defined among non-diabetics as any of HbA1c ≥5.6% and <6.5% or fasting plasma glucose ≥5.6 mmol/L and <7 mmol/L or prandial plasma glucose ≥7.8 mmol/L and <11.1 mmol/L.

Isolated IFG = isolated impaired fasting glucose; Isolated IGT = isolated impaired glucose tolerance.
