# Supplementary figures and images for: Association Between Mycobacterium tuberculosis Sensitization and Insulin Resistance Among US Adults Screened for Type 2 Diabetes Mellitus
Source: Open Forum Infect Dis. 2024 Oct 28;11(10):ofae568. doi: 10.1093/ofid/ofae568 (PMC11518572; doi:10.1093/ofid/ofae568)

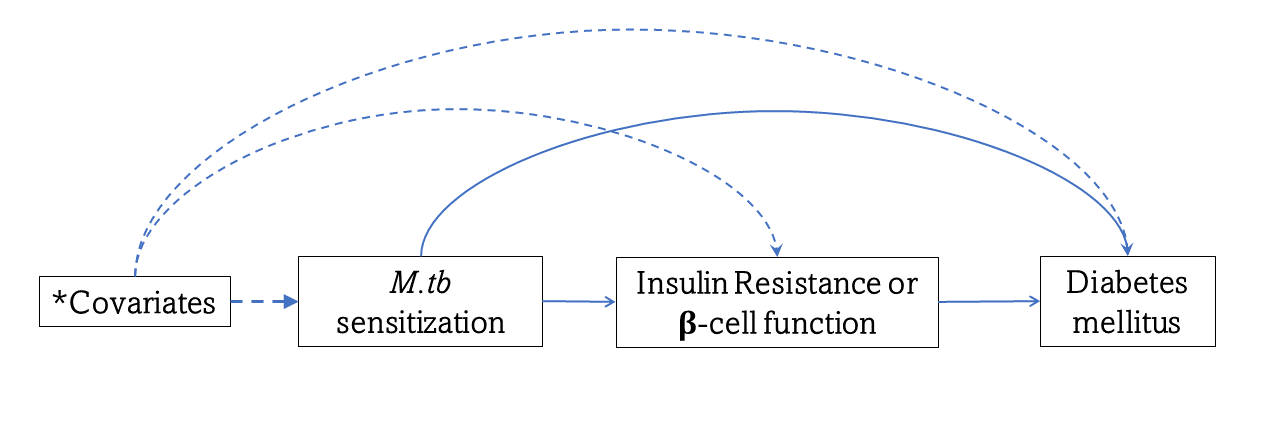

Supplement: ofae568_Supplementary_Data [file ofae568_supplementary_data.zip › Supplementary Figure 1.png]
